# Regenerative Potential of Blood-Derived Products in 3D Osteoarthritic Chondrocyte Culture System

**DOI:** 10.3390/cimb43020048

**Published:** 2021-07-11

**Authors:** Olga Kuten-Pella, Andrea De Luna, Karina Kramer, Markus Neubauer, Stefan Nehrer, Zsombor Lacza

**Affiliations:** 1 OrthoSera GmbH, 3500 Krems, Austria; lacza.zsombor@tf.hu; 2 Center for Regenerative Medicine, Danube University Krems, 3500 Krems, Austria; andrea.deluna@donau-uni.ac.at (A.D.L.); karina.kramer@donau-uni.ac.at (K.K.); markus.neubauer@donau-uni.ac.at (M.N.); stefan.nehrer@donau-uni.ac.at (S.N.); 3 Institute of Sport and Health Sciences, University of Physical Education, H-1123 Budapest, Hungary

**Keywords:** osteoarthritic chondrocytes, cartilage, 3D pellet culture, blood products, platelet-rich plasma, hyperacute serum

## Abstract

Intra-articular injection of different types of blood-derived products is gaining popularity and clinical importance in the treatment of degenerative cartilage disorders such as osteoarthritis. The regenerative potential of two types of platelet-rich plasma (PRP), prepared in the presence of EDTA (EPRP) and citrate (CPRP) and an alternative blood product-hyperacute serum (hypACT) was evaluated using a 3D osteoarthritic chondrocyte pellet model by assessing the metabolic cell activity, cartilage-related gene expression and extracellular matrix deposition within the pellets. Chondrocyte viability was determined by XTT assay and it revealed no significant difference in metabolic activity of OA chondrocyte pellets after supplementation with different blood products. Nevertheless, the selection of blood products influenced the cartilage-related genes expression, ECM morphology and the tissue quality of pellets. Both PRP types had a different biological effect depending upon concentration and even though CPRP is widely used in clinics our assessment did not reveal good results in gene expression either tissue quality. HypACT supplementation resulted in superior cartilage-related genes expression together with tissue quality and seemed to be the most stable product since no remarkable changes were observed between the two different concentrations. All in all, for successful regenerative therapy, possible molecular mechanisms induced by blood-derived products should be always carefully investigated and adapted to the specific medical indications.

## 1. Introduction

Osteoarthritis (OA) is a common form of degenerative, joint destructive disease affecting 70% of the population beyond 70, where up to 30% of the cases are symptomatic. Due to demographic changes, this number is likely to increase [1,2]. The underlying pathophysiology of OA is multifactorial, however, the combination of factors such as age, progressive cartilage degeneration and joint inflammation seems to be crucial to its etiology [3]. Minor cartilage injuries may heal on their own within a few weeks, but full-thickness defects have a poor capacity for repair and it may lead to OA [4,5]. Regenerative therapy is a novel strategy to stimulate proliferation and matrix synthesis of chondrocytes and neighbouring cells to restore a normal structure and function of damaged cartilage. Different studies have proposed the use of platelet derivatives in order to facilitate the repair system of bones and cartilages [6]. In vitro experiments showed that platelet-rich-plasma (PRP) supplementation enhances proliferation, migration and matrix biosynthesis of articular chondrocytes [7].

However, heterogeneous production protocols lead to discrepancies in PRP standardization and due to the different composition, it is difficult to predict the effects of a treatment strategy. PRP was reported to contain pro-inflammatory components such as leucocytes or fibrin [8,9] the presence of which might be unfavourable in the case of an inflamed joint. This demonstrates the necessity for an extensive understanding of the underlying biologic mechanisms of blood-derived products action. Hyperacute serum (hypACT) is a PRP alternative, cell- and platelet-free blood product without fibrin matrix [10]. It was shown that chondrocytes supplemented with hypACT (previously called HAS) proliferate faster even when affected by osteoarthritis. Addition of hypACT to adipose-derived stem cells cultured in 3D pellets together with chondrogenic differentiation medium resulted in obtaining pellets with good tissue quality and enhanced expression of cartilage-related genes. However, our previous studies evaluating hypACT and PRP influence on OA chondrocytes behaviour were limited to monolayer culture experiments which may not adequately mimic the in vivo conditions [11,12,13].

This study aimed to (i) evaluate and (ii) compare the effects of different blood-derived products: hypACT and two types of PRP upon their regenerative potential assessed by cell proliferation and matrix synthesis in an established 3D OA chondrocyte in-vitro culture system.

## 2. Materials and Methods

### 2.1. The Preparation of Blood Products

Blood products: hypACT, leukocyte-poor PRP prepared from blood anticoagulated with EDTA (EPRP) and leukocyte-poor PRP prepared from blood anticoagulated with trisodium citrate (CPRP) were produced from whole blood collected from 9 healthy donors (5 female, 4 men; age between 25 and 45 years old). During the collection around 50 mL of whole blood was drawn from each donor. The following volumes of blood were used: 16 mL for EPRP, 16 mL for CPRP and 18 mL for hypACT. Each blood product was prepared by methods described by Neubauer, Kuten et al. [12]. All nine samples of each product were pooled and immediately used for experiments or stored at −80 °C for later use. Written informed consent was obtained from all donors.

### 2.2. OA Chondrocytes Isolation

Human osteoarthritic articular cartilage was obtained from 5 patients (3 female, 2 male) who had an average of 73 years and underwent total knee arthroplasty. Chondrocyte isolation and expansion protocols were previously described by Bauer et al. [13]. Written informed consent was obtained from all donors. Ethical approval for the use of OA chondrocytes was obtained from the Ethics Committee of Lower Austria (GS1-EK-4/480-2017).

### 2.3. OA Chondrocytes Pellet Culture

For 3D pellet cell culture model Gibco DMEM high glucose medium supplemented with: GIBCO^®^ DMEM GlutaMAX™ Supplement, pyruvate (Invitrogen, LifeTech Austria, Vienna, Austria), 1% ITS (Sigma-Aldrich Chemie GmbH, Steinheim, Germany), 100 nM dexamethasone (Sigma-Aldrich Chemie GmbH, Steinheim, Germany), 50 µg/mL ascorbic acid, 1% non-essential amino acids (Gibco Life Technologies Europe Bv, Bleiswijk, The Netherlands), 5 ng/mL TGFbeta-3 (PeproTech, New York, NY, USA), 4% methyl cellulose (Sigma-Aldrich, St. Louis, MI, USA) was used with the addition of 1% or 5% different blood product supplementation or FCS. 250,000 of OA chondrocytes expanded to passage 1 were mixed with chondrogenic media and placed in 15 mL polypropylene tubes. To form the pellets samples were centrifuged at the highest speed 4164× *g* for 10 min. The supplementation of pellets lasted 3 weeks and the culture medium was changed every three days.

### 2.4. Metabolic Activity Assay (XTT Assay)

On day 21 of pellet culture, OA chondrocytes pellets were transferred together with 100 µL of their culture medium to 96-well plates. Metabolic activity of the OA chondrocytes was investigated by the XTT assay (Roche Diagnostics GmbH, Mannheim, Germany) according to the manufacturer’s protocol. Relative absorbance was measured by using a plate reader (BioTek’s Synergy 2, Winooski, VT, USA).

### 2.5. RNA Extraction and Quantitative Real-Time Polymerase Chain Reaction (qRT-PCR)

RNA Extraction: After 21 days of pellet culture, three OA chondrocyte pellets per blood product were collected in 200 µL PBS. Pellets were homogenized by adding the ceramic beads in a MagnaLyser device (MagNA Lyser Green Beads, Roche Diagnostics, Basel, Switzerland). The homogenization (6500 rpm, 20 s) was repeated twice with a 2 min cooling phase in between (8–12 °C). The RNA was isolated using the High Pure RNA Isolation kit (Roche Diagnostics GmbH, Mannheim, Germany) according to the manufacturer’s protocol. RNA was eluted and stored at −80 °C until cDNA synthesis. 

Gene Expression Analysis: cDNA-Synthesis was performed using Transcriptor First Strand cDNA Synthesis Kit (Roche, Basel, Switzerland). Real-time quantitative polymerase chain reaction (RTqPCR) was performed in triplicates using the LightCycler^®^ 96 from Roche. In total, five genes: Collagen type 2 (*COL2AB*), Collagen type 1 (*COL1A1*), *SOX9*, Matrix Metalloproteinase-3 (*MMP3*) and cyclin-dependent kinase inhibitor 1 (*P21*) were analyzed. The sequences of the used primers are presented in Table 1. Glyceraldehyde-3-phosphate dehydrogenase (*GAPDH*) was used as a housekeeping gene. 

### 2.6. Statistical Analysis

Statistical analysis was performed using GraphPad Prism version 8.0.0 for Windows, GraphPad Software (San Diego, CA, USA). Metabolic activity and PCR results were analyzed by using Kruskal–Wallis combined with the Dunns post-test. *p* < 0.05 was considered significant. Data are presented as mean  ±  SEM.

## 3. Results

### 3.1. There Is No Significant Difference in Metabolic Activity between the OA Chondrocytes from Pellet Culture with Different Blood Products

**Figure 1:**
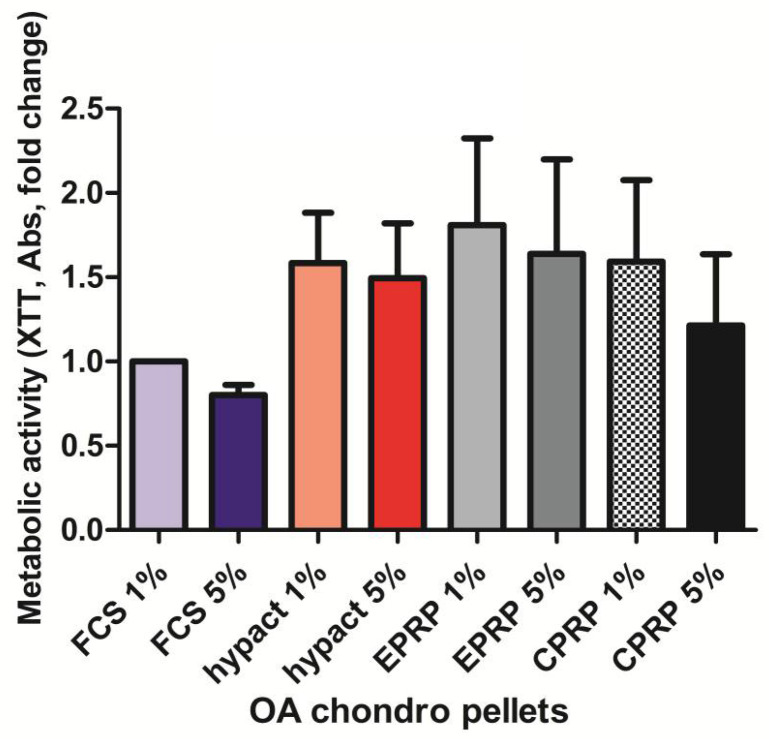
Metabolic activity of OA chondrocytes in 3D pellet culture, *n* = 5 (CPRP = citrate platelet-rich plasma, EPRP = EDTA platelet-rich plasma, FCS = fetal calf serum, hypACT = hyperacute serum, Results are expressed as the mean  ±  SD).

After 21 days of pellets culture, the OA chondrocytes supplemented with 1% or 5% of hypACT, EPRP and CPRP, did not reveal any significant changes in metabolic activity versus the control group supplemented with 1% FCS (Figure 1). The values for hypACT, EPRP and 1% CPRP were approximately 1.5 times higher than FCS 1% group. For 5% CPRP only a slight 1.2 fold increase was reported and FCS 5% was the only group where chondrocytes had lower metabolic activity than the control group.

### 3.2. Blood Products Influence the Chondrogenic Genes Expression

**Figure 2:**
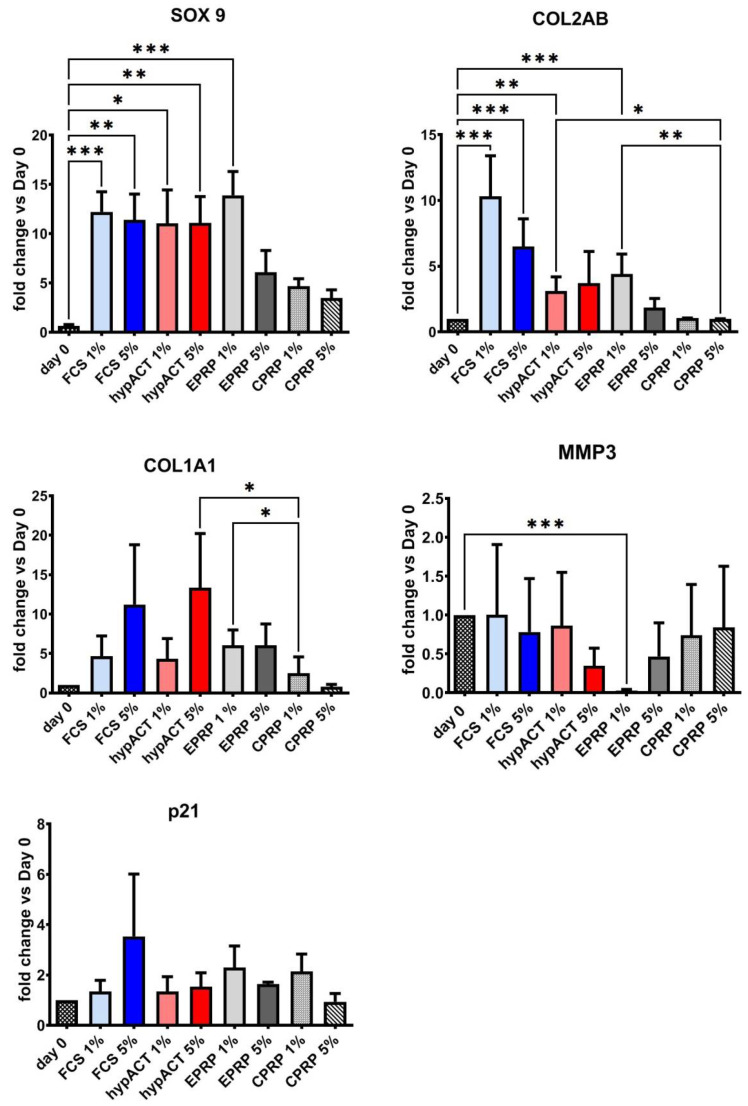
Gene expression levels in OA chondrocytes from 3D pellet culture, *n* = 5 (*COL1A1* = collagen 1A1 gene, *COL2AB* = collagen 2 gene, CPRP = citrate platelet-rich plasma, EPRP = EDTA platelet-rich plasma, FCS = fetal calf serum, hypact = hyperacute serum, *MMP3* = matrix-metalloproteinase 3 gene), *P21* = cyclin-dependent kinase inhibitor-1A, *SOX9* = transcription factor *SOX9*. Results are expressed as the mean  ±  SD. * represents *p*  <  0.05, ** represents *p*  <  0.01, and *** represents *p*  <  0.001. To improve transparency of presented data asterisks for significant changes of FCS 1% and 5% versus other blood products have been omitted (included in Table 2).

Differences in gene expression levels (typical chondrogenic-related genes: *SOX9*, *COL2AB* and additional genes: *MMP3*, *COL1A1* and *P21*) were analyzed after 21 days of pellets culture with regards to the type of supplementation (blood products or FCS) and the used concentration (1% or 5%) (Figure 2). Day 0 represent the gene expression of OA chondrocytes in 2D cell culture before the shift to 3D pellet culture and supplementation with blood products. 

General increase of *SOX9* expression was observed in all tested groups after 21 days of culture. However, significant change was reported only versus day 0 in groups supplemented with FCS 1% (*p* = 0.0007), FCS 5% (*p* = 0.0046), hypACT 1% (*p* = 0.0169), hypACT 5% (*p* = 0.0043) and EPRP 1% (*p* = 0.0002). Chondrocytes supplemented with EPRP 5%, CPRP 1% and CPRP 5% expressed lower levels of *SOX9* which were not significant. Expression of another cartilage biomarker *COL2AB* was significantly higher versus day 0 in FCS 1% (*p* < 0.001), FCS 5% (*p* < 0.001), hypACT 1% (*p* = 0.004), and EPRP 1% (*p* < 0.001) groups. A nonsignificant trend was identified in hypACT 5% group (*p* = 0.09). No increase in *COL2AB* production was observed in chondrocytes supplemented with CPRP 1% and 5% and it was significantly lower than in groups with FCS 1% (*p* = 0.01; *p* < 0.001) and 5% supplementation (*p* = 0.03; *p* < 0.001). Additionally, hypACT 1% and EPRP 1% had significantly higher *COL2AB* level than CPRP 1% (*p* = 0.01, *p* = 0.003). *COL1A1* which is a hypertrophic differentiation marker [14] was especially upregulated in hypACT 5% group and the observed increase was significant versus CPRP 1% (*p* = 0.02) as well as in case of EPRP 1% versus CPRP 1% group (*p* = 0.003). Interestingly, supplementation with 1% EPRP resulted in an extensive drop of *MMP3* expression versus day 0 (*p* < 0.001), whereas the addition of hypACT 5% and EPRP 5% caused only a moderate, not significant decrease vs. day 0. Additionally, a senescent marker *p21* was included in the study. The *p21* expression was higher only in OA chondrocytes which were treated with 5% FCS but there was no significant difference versus day 0 and it remained stable in all the other groups.

### 3.3. Blood Products Influence ECM Morphology and the Tissue Quality of Chondrocyte Pellets

**Figure 3:**
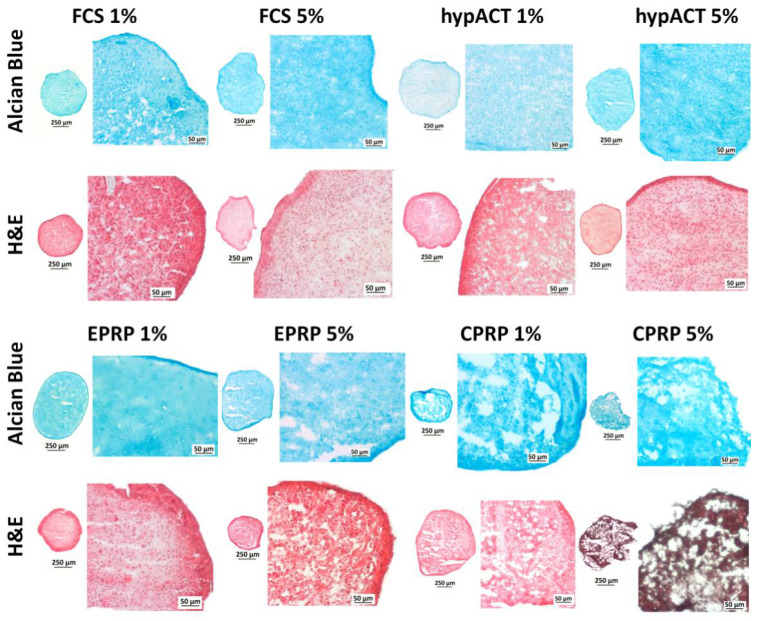
Histology results (CPRP = citrate platelet-rich plasma, EPRP = EDTA platelet-rich plasma, FCS = fetal calf serum, H&E = hematoxylin and eosin, hypACT = hyperacute serum).

The histological cuts after 21 days of pellet culture are presented in Figure 3. These microscopic pictures show a qualitative assessment and the histological appearance differs in between groups. 

Histological slices derived from pellets supplemented with FCS 5%, hypACT 5% and EPRP 1% show a confined ECM morphology and good tissue quality. Samples from FCS 1%, hypACT 1% and EPRP 5% show a diffuse but homogenous ECM morphology and average tissue quality, however, based on this qualitative assessment the pellets from EPPR 5% group, seem to have slightly more fissures than FCS 1% and hypACT 1%. Cuts from 1% and 5% CPRP groups show a diffuse and heterogenous ECM composition with poor tissue quality. Many fissures, disintegrated tissue islands and platelets clumps can be found especially in pellets supplemented with 5% CPRP.

## 4. Discussion

The aim of this study was to compare hypACT and PRP efficacy in the treatment of cartilage degeneration utilizing 3D culture model. During OA progression chondrocytes acquire a degenerated hypertrophic phenotype similar to that reported in experimental OA in vitro models [14,15,16,17,18]. In 2D cell culture system chondrocytes tend to dedifferentiate and adopt an elongated fibroblast-like shape that has been associated with an altered genetic profile, including a reduction in the expression of cartilage-specific genes [19]. In recent years, several studies have demonstrated the redifferentiation potential of OA chondrocytes can be induced by platelet-rich plasma [20,21,22]. It seems that PRP can influence the cell-hostile microenvironment of inflammation or cell disintegration and to slow or even reverse the degenerative process. The presence of specific growth factors and cytokines is contributing to improvement of cell survival, reduced inflammation and restoration of the whole knee joint homeostasis. As previously described by Kardos et al. the overall growth factor and cytokine content in PRP is higher than in hypACT with few exceptions, including the level of bFGF. Examples of growth factors highly enriched in PRP, but not in hypACT are PDGF, VEGF or TGF-β [10,23]. TGF-β supports tissue regeneration such as bone formation [24] or cartilage development [25,26], however, it may also participate in tissue degradation [27,28,29] and was categorized not only as an anti-inflammatory but also as a pro-inflammatory cytokine, depending on the circumstances [30,31]. Therefore, the concentration of not only TGF-β1 but various growth factors beyond critical levels may have serious side effects [32,33] and it is not easy to predict the possible biological effects of highly enriched blood products such as PRP. 

In previous studies, we observed that hypACT is supporting MSCs, osteoblasts and OA chondrocytes proliferation in monolayer culture [11,12,34]. However, 2D culture system seems to be inadequate for cartilage regeneration and 3D models are more effective in mimicking chondrocyte physiological conditions. For this reason, a chondrocyte pellet culture was implemented in this study. A similar model was previously used by De Luna et al. and was shown to be feasible for basic OA-related research questions [35]. To assess the best supplementation for pellets, initially, we tested the same culture conditions as for monolayer culture. Although, we observed that 10% of blood products or FCS supplementation, is not suitable for pellet culture and leads to very poor tissue quality within the pellets. Consequently, lower blood products concentration: 1% and 5% were used in this study. Surprisingly, when metabolic activity of cells in pellets was measured the results were divergent from these previously observed in 2D culture. Therefore, the hypACT supplementation in the 3D system did not manifest as beneficial effects as in monolayers. However, the XTT assay used for pellet culture may have some limitations regarding the pellet penetration with reagents depending on pellet morphology and size. It should be noted that these results may not reflect the viability of chondrocytes within the pellet core, but only on the pellet surface. 

According to the literature the comparison of transcript levels from normal and OA cartilage samples reveals a substantial reduction of *SOX9* and *COL2AB*, while production and activity of MMPs are highly increased during the OA development. These changes lead to the loss of matrix and gradual fail of cartilage biological function [36,37,38]. The same expression pattern is also observed during the dedifferentiation of chondrocytes undergoing in vitro expansion [36,39]. Therefore it may be very challenging to distinguish whether the chondrocyte phenotype changes in 2D systems are involved with OA pathology, inadequate supplementation or unfavourable culture conditions. 

Regarding the levels of *SOX9* and *COL2AB* in our culture system, 3D pellet culture together with the chondrogenic differentiation media led to significantly increased expression vs. day 0 in the groups with FCS, hypACT and 1% EPRP. *SOX9* expression reached almost the same level in the mentioned groups, whereas the differences in *COL2AB* between these groups were much more exposed. These PCR results correlate with histological analysis where the best tissue quality was obtained after the supplementation with FCS, hypACT and 1% EPRP. Based on the known features of chondrocytes cultured in monolayers we expected a higher expression of *COL1A1* on day 0 and a drop after implementing pellet culture and blood product supplementation. Surprisingly, an overall increase of *COL1A1* expression was observed (despite the 5% CPRP group) with the highest values in the 5% FCS and 5% hypACT groups. An explanation for this effect could be a relatively diminished level of *MMP3* in 5% hypACT, since the level of collagens within cartilage depends on the action of matrix metalloproteinases [37], nevertheless, this observation is not consistent with results from 1% EPRP group. 1% EPRP treatment showed a significant drop of *MMP3* vs. day 0 and was not observed in any other group. At the same time the level of *COL1A1* in 1% EPRP was not increased as expected, but almost the same as in 5% EPRP, 1% FCS and 1% hypACT. Interestingly, when we compared the expression of senescent marker *p21* with XTT results, the lowest metabolic activity in the 5% FCS group was reflected with the highest expression of *p21* however, it was a nonsignificant trend. This observation was not correlated with the histology of pellets supplemented with 5% FCS, which exposed good tissue quality and no signs of disturbed cell morphology.

Comparison of results from ECM production indicates that the most consistent data were obtained in EPRP and hypACT groups. Analyzing the glycosaminoglycans deposition within the pellets EPRP 1% rather than 5% seems to be a favouring supplementation. 5% EPRP resulted in higher platelet accumulation within the pellet structure and slightly lower ECM quality. HypACT supplementation results in superior cartilage-related genes expression together with tissue quality and it seems to be the most stable product since no remarkable changes were observed between the two concentrations. In the case of the cells supplemented with 1% and 5% CPRP microscopic examination of pellet cuts showed poor and very poor ECM production, high platelets accumulation and diminished chondrocytes presence. Not only histological results revealed a negative effect of CPRP supplementation in the 3D system, but also the expression of cartilage related genes was lower in the CPRP group when compared to other blood products. At the same time based on the results from XTT assay CPRP treatment should not lead to such unfavourable effects as revealed in histology and gene expression. However, relatively high metabolic activity might be explained by extensive platelet accumulation after 21 days of cultivation with 5% CPRP. 

The observed discrepancy in the chondrocytes behaviour between EPRP and CPRP vs. hypACT and FCS may be involved with the high, non-physiological concentration of growth factors in PRPs as well as the presence of anticoagulants (citrate, EDTA) during its preparation. The need for the addition of anticoagulants to PRP is a serious disadvantage since anticoagulants significantly reduce the natural coagulation cascade, thereby, may influence some physiological processes [40]. Even though EPRP is not used in clinical settings due to its known negative effects on cell viability [11,34] it showed superiority regarding tissue quality and gene expression in comparison to CPRP. A possible mechanism explaining these differences might be the chelating feature of EDTA, which traps free calcium ions and prevents chondrogenesis inhibition [41]. Likewise, the “untrapped” free calcium ions in the case of CPRP might explain the inferior chondrogenic results both in histology as well as in gene expression. Additionally, the accumulation of platelets within OA chondrocyte pellets supplemented with CPRP might alter the forming tissue quality. Even though CPRP is widely used in clinics our assessment did not reveal good results in gene expression either in tissue quality and these observations are in line with our previous study [12]. It is also important that because of the animal origin of FCS, this supplementation should only be considered as a positive control group without possible clinical application.

## 5. Conclusions

Based on our previous studies as well as available literature reports we observed that utilizing only 2D in vitro cultures is not enough to predict the biological effects of blood products. More complex 3D and explants models should be designed for a better understanding of their role in tissue homeostasis and repair. Our OA chondrocyte pellet culture model was established to enlarge the basic knowledge and to compare the previously investigated blood products such as CPRP, EPRP and hypACT and their potential clinical use. After analyzing different aspects we could observe some similarities among the supplementation groups, however, characteristic features for every treatment were always reported. Therefore, the selection of a blood product should be always carefully considered and supported by relevant research before it is used for any therapy. In our opinion, successful treatment depends on the selection of the product with the most favourable growth factors composition as well as their concentration. Ideally, future regenerative therapies with blood-derived products should be carefully controlled and customized to the specific medical condition and patient individual requirements.

## 6. Patents

Z.L. and O.K.-P. are inventors in a granted patents for fabrication of serum fraction of platelet-rich fibrin (US20140227241A1), a new method for increasing proliferation rate of chondrocytes (WO2017152172A1) and a method of preparing an isolated serum fraction of platelet rich fibrin and its use as a cell culture additive (US20190167723A1).

## Figures and Tables

**Table 1:** Primers used in quantitative real-time polymerase chain reaction.

Markers
*GAPDH* FORWARD: 5′-CTCTGCTCCTCCTGTTCGAC-3′ REVERSE: 5′-ACGACCAAATCCGTTGACTC-3′
*COL1A1* FORWARD: 5′-GGGATTCCCTGGACCTAAAG-3′ REVERSE: 5′-GGAACACCTCGCTCTCCAG-3′
*COL2AB* FORWARD: 5′-GCACCTGCAGAGACCTGA-3′ REVERSE: 5′-GGGTCAATCCAGTAGTCTCCAC-3′
*SOX9* FORWARD: 5′-TACCCGCACTTGCACAAC-3′ REVERSE: 5′-TCTCGCTCTCGTTCAGAAGTC-3′
*MMP3* FORWARD: 5′-CAAAACATATTTCTTTGTAGAGGACAA-3′ REVERSE: 5′-TTCAGCTATTTGCTTGGGAAA-3′
*P21* FORWARD: 5′-TCACTGTCTTGTACCCTTGTGC-3′ REVERSE: 5′-GGCGTTTGGAGTGGTAGAAA-3′

**Table 2:** Significant differences in gene expression between supplementation groups.

Gene	Dunn’s Multiple Comparisons Test	Summary	Adjusted *p* Value
*SOX9*	day 0 vs. FCS 1%	***	0.0007
	day 0 vs. FCS 5%	**	0.0046
	day 0 vs. hypACT 1%	*	0.0169
	day 0 vs. hypACT 5%	**	0.0043
	day 0 vs. EPRP 1%	***	0.0002
*COL2AB*	day 0 vs. FCS 1%	***	<0.001
	day 0 vs. FCS 5%	***	<0.001
	day 0 vs. hypACT 1%	**	0.0040
	day 0 vs. EPRP 1%	***	<0.001
	FCS 1% vs. CPRP 1%	*	0.0100
	FCS 1% vs. CPRP 5%	***	<0.001
	FCS 5% vs. CPRP 1%	*	0.0300
	FCS 5% vs. CPRP 5%	***	<0.001
	hypACT 1% vs. CPRP 5%	*	0.0100
	EPRP 1% vs. CPRP 5%	**	0.0030
*COL1A1*	hypACT 5% vs. CPRP 1%	*	0.0200
	EPRP 1 % vs. CPRP 1%	*	0.0200
*MMP3*	day 0 vs. EPRP 1%	***	<0.001

* represents *p*  <  0.05, ** represents *p*  <  0.01, and *** represents *p*  <  0.001.
